# Dengue Virus Serotype 2 Blocks Extracellular Signal-Regulated Kinase and Nuclear Factor-κB Activation to Downregulate Cytokine Production

**DOI:** 10.1371/journal.pone.0041635

**Published:** 2012-08-22

**Authors:** Tsung-Hsien Chang, Siang-Ru Chen, Chia-Yi Yu, You-Sheng Lin, Yao-Shen Chen, Toru Kubota, Mayumi Matsuoka, Yi-Ling Lin

**Affiliations:** 1 Department of Medical Education and Research, Kaohsiung Veterans General Hospital, Kaohsiung, Taiwan; 2 Institute of Biomedical Sciences, Academia Sinica, Taipei, Taiwan; 3 Department of Biological Sciences, National Sun Yat-Sen University, Kaohsiung, Taiwan; 4 Section of Infectious Diseases, Department of Internal Medicine, Kaohsiung Veterans General Hospital, Kaohsiung, Taiwan; 5 Section of Microbiology, Department of Pathology and Laboratory Medicine, Kaohsiung Veterans General Hospital, Kaohsiung, Taiwan; 6 Department of Virology III, National Institute of Infectious Diseases, Tokyo, Japan; 7 Department of Bacteriology II, National Institute of Infectious Diseases, Tokyo, Japan; French National Centre for Scientific Research, France

## Abstract

**Background:**

Dengue virus (DENV) infection is the most common mosquito-borne viral disease threatening human health around the world. Type I interferon (IFN) and cytokine production are crucial in the innate immune system. We previously reported that DENV serotype 2 (DENV-2) induced low levels of interferon regulatory factor 3 and NF-κB activation, thus leading to reduced production of IFN-β in the early phase of infection. Here, we determined whether DENV infection not only hampers type I IFN activation but also cytokine production triggered by Toll-like receptor (TLR) signaling.

**Methodology/Principal Findings:**

We used quantitative RT-PCR and found that only low levels of IFN-β and inflammatory cytokines such as interleukin 10 (IL-10), IL-12 and tumor necrosis factor α (TNFα) mRNA were detected in DENV-2–infected bone-marrow–derived dendritic cells. Furthermore, DENV-2 infection repressed cytokine production triggered by TLR signaling. To elucidate the molecular mechanisms underlying this suppression event, we measured NF-κB activation by p65 nuclear translocation and luciferase reporter assay and found that NF-κB activation triggered by TLR ligands was blocked by DENV-2 infection. As well, extracellular signal-regulated kinase (ERK) activity was suppressed by DENV-2 infection.

**Conclusions/Significance:**

To downregulate the host innate immunity, DENV-2 by itself is a weak inducer of type I IFN and cytokines, furthermore DENV-2 can also block the TLR-triggered ERK–NF-κB activation and cytokine production.

## Introduction

Dengue virus (serotypes DENV-1, -2, -3 and -4) is a positive-strand RNA virus belonging to the family *Flaviviridae*, genus *Flavivirus*. Mosquitoes transmitting DENV in humans has been a major cause of dengue diseases in tropical and subtropical counties; approximately one-third of the world's population is at risk of the infection. People infected with DENV typically show self-limited febrile dengue fever and dengue hemorrhagic fever (DHF). Life-threatening dengue shock syndrome (DSS) is more likely to occur after a second DENV infection [1], [2]. Other than supportive treatments, no specific therapy is available for dengue-related diseases. Several tetravalent DENV vaccine candidates are under development, but an effective, safe and affordable dengue vaccine remains elusive [3].

DENV infects multiple organs and cell types in humans. Particularly, the mononuclear phagocyte lineage of macrophages, monocyte-derived dendritic cells (DCs) and skin Langerhans cells are the primary cell targets [4], [5], [6]. Similar cellular tropism of macrophages and DCs was observed in the experimental DENV mouse infection model [7]. Macrophages and DCs are the most crucial cell types in innate immunity and rapidly produce type I interferons (IFNs) and cytokines to fight against microbe invasion. Type I IFNs are potent inhibitors of virus replication. Therefore, many pathogenic viruses have developed strategies to escape the IFN-triggered anti-viral effects. More than 170 different virus-encoded IFN antagonists from 93 distinct viruses have been described [8]. For example, hepatitis C virus (HCV), a member of *Flaviviridae*, evades innate immunity by cleaving mitochondrial antiviral signaling protein, an IFN stimulator, with its protease NS3/4A [9]. The nonstructural proteins NS4B of DENV-2, West Nile virus (WNV), and yellow fever virus (YFV) block the activation of STAT1 in cells stimulated with type I IFN [10], [11].

We previously found that two flaviviruses, Japanese encephalitis virus and DENV-2, trigger type I IFN transcription through an RIG-I-dependent signaling cascade to activate interferon regulatory factor (IRF) and NF-κB. However, JEV induced higher activation of IRF3 and NF-κB than DENV-2 in human A549 cells [12]. Furthermore, type I IFN production triggered by double-stranded RNA (dsRNA) stimulation was blocked in DENV-2-infected human DCs [13], [14]. Microarray results from rhesus macaques also indicated that type I IFN, interleukin 10 (IL-10), IL-8, IL-6 and tumor necrosis factor α (TNFα) were not upregulated with DENV-1 infection [15]. Therefore, DENV-2 might modulate the induction pathway of type I IFN and other cytokines.

The Toll-like receptor (TLR) family is one of the best-studied pattern-recognition receptor families and is responsible for sensing invading pathogens [16]. Different TLRs recognize the different molecular patterns of microorganisms; for example, TLR4 recognizes lipopolysaccharide (LPS) and TLR3 recognizes dsRNA. Engagement of TLRs with their ligands triggers signal cascades to activate IRFs and NF-κB, thus leading to production of cytokines and type I IFN [17]. NF-κB activation is crucial for cytokine induction, and many viruses evolve various strategies to manipulate NF-κB signaling [18], [19]. Several RNA virus-encoded proteins, such as HCV NS5B, SARS CoV M protein, measles virus V protein, and enterovirus 71 2C, inhibit NF-κB activation directly or indirectly [20], [21], [22], [23].

In this study, we investigated whether DENV-2 could block type I IFN and cytokine induction triggered by TLR signaling. We studied the influence of DENV-2 infection on activation of NF-κB and extracellular signal-regulated protein kinase (ERK).

## Materials and Methods

### Virus, cell lines, chemicals and antibodies

The DENV-2 PL046 strain (Genbank accession: AJ968413.1) was isolated from a Taiwanese DF patient. The DENV-2 prototype New Guinea C (NGC) strain was kindly provided by D. J. Gubler of the Centers for Disease Control and Prevention, USA. These viruses were propagated in the mosquito cell line C6/36 (ATCC: CRL-1660) grown in RPMI 1640 medium containing 5% fetal bovine serum (FBS) [24]. The J774A.1 mouse macrophage cell line (ATCC: TIB-67), A549 human lung epithelial carcinoma cell line (ATCC: CCL-185), and African green monkey kidney epithelial cell line Vero (ATCC: CCL-81) were cultured in DMEM medium supplemented with 10% FBS (Invitrogen). The TLR3 ligand polyinosine-polycytidylic acid (polyI:C) and TLR9 ligand CpG oligodeoxynucleotides 1826 (CpG ODN 1826; hereafter CpG) were from InvivoGen. The TLR4 ligand LPS (Sigma-Aldrich), anti-ERK antibody, anti-phospho-ERK antibody (Cell Signaling, catalog# 9102 and 9101S) and anti-NF-κB p65 antibody (sc-372, Santa Cruz Biotechnology) were used.

### Bone-marrow–derived DC (BMDC) cultures and stimulation

Female C57BL/6 mice at 6–8 weeks old were used in accordance with the guidelines of Kaohsiung Veterans General Hospital animal care and use committee under the approved animal study protocol (VGHKS-99-A028). BMDCs were generated by culturing bone-marrow hematopoietic cells with FMS-like tyrosine kinase 3 ligand (Flt3L) for 8 days [25], [26]. For DENV-2 infection, 10^6^ DCs were adsorbed with DENV-2 at a multiplicity of infection (MOI) of 5 for 1 h. After removing the virus inoculant, cells were incubated with complete medium. For stimulation with TLR ligands, DCs were incubated with 100 µg/ml polyI:C or 1 µg/ml CpG.

### Real-time quantitative PCR (qPCR) and primers

TRIzol reagent (Invitrogen) was used for total RNA extraction, and cDNA was synthesized from 0.5 µg total RNA by Superscript III reverse transcriptase (Invitrogen). qPCR amplification was done with 4 ng cDNA in 10 µl SYBR Green PCR master mix (Applied Biosystems) with 3 µM of primers in the ABI Prism 7000 Sequence Detection System (Applied Biosystems). Transcript levels were normalized to that of hypoxanthine phosphoribosyltransferase (HPRT). The primer pairs were for *IFN-β*, 5′-GCTCCTGGAGCAGCTGAATG-3′ and 5′-CGTCATCTCCATAGGGATCTTGA-3′; *IL-10*, 5′-GATGCCCCAGGCAGAGAA-3′ and 5′-CACCCAGGGAATTCAAATGC-3′; *TNFα*, 5′-CACAAGATGCTGGGACAGTGA-3′ and 5′-TCCTTGATGGTGGTGCATGA-3′; *IL-12p40*, 5′-ACAGCACCAGCTTCTTCATCAG-3′ and 5′-TCTTCAAAGGCTTCATCTGCAA-3′; *HPRT*, 5′-GCTCGAGATGTCATGAAGGAGAT-3′ and 5′-AAAGAACTTATAGCCCCCCTTGA-3′; DENV-2 *5*′*UTR* 5′- AGTTGTTAGTCTACGTGGACCGA-3′ and 5′-CGCGTTTCAGCATATTGAAAG-3′. The qPCR primers for TLRs are listed in Table S1.

### Immunofluorescence assay

Cells were fixed with 4% paraformaldehyde for 30 min, then permeabilized with 0.5% Triton X-100 for 10 min. After 2 washes with PBS, cells were blocked with 10% skim milk in PBS. NF-κB p65 subcellular location was determined by immunostaining with rabbit anti-NF-κB p65, then Alexa Fluor-568-conjugated goat anti-rabbit IgG antibody (Invitrogen). DENV-2 NS3 was detected by a specific monoclonal antibody against NS3 (#YH3304, 1∶500 dilution, Yao-Hong Biotechnology) plus Alexa Fluor-488-conjugated goat anti-mouse IgG antibody (Invitrogen). Fluorescence signals were observed under a fluorescence microscope (Olympus BX51).

### Luciferase reporter assay

TurboFect transfection reagent (Fermentas) was used for transient transfection following the manufacturer's protocol. Cells cultured in 12-well plate were transfected with NF-κB- or IFN-β-Luc reporter plasmids [12], [25]. pRL-TK (Promega), encoding *Renilla* luciferase under an HSV thymidine kinase promoter, was used as an internal control. After transfection for 24 h, cells were infected with DENV-2; in some experiments, cells were further stimulated with LPS or polyI:C (both 1 µg/ml). Cell lysates were collected at the indicated times for dual-luciferase assays (Promega). Relative firefly luciferase activity was normalized to *Renilla* luciferase activity.

### Immunoblot analysis

Cells were lysed in RIPA buffer (150 mM NaCl, 0.5% sodium deoxycholate, 1% NP40, 0.1% SDS, 50 mM Tris-HCl [pH 8.0]) containing protease inhibitor and phosphatase inhibitor cocktails (Roche). Harvested cell extracts were separated by 10% SDS-PAGE and transferred to PVDF membranes, which were reacted with primary antibody, and then horseradish peroxidase-conjugated secondary antibody (Jackson ImmunoResearch Laboratory) and visualized with an enhance chemiluminescence system (Thermo). Images were acquired by a digital image system (UVP or Fujifilm).

## Results

### Low levels of IFN-β and cytokine production in DENV-2–infected BMDCs

**Figure 1:**
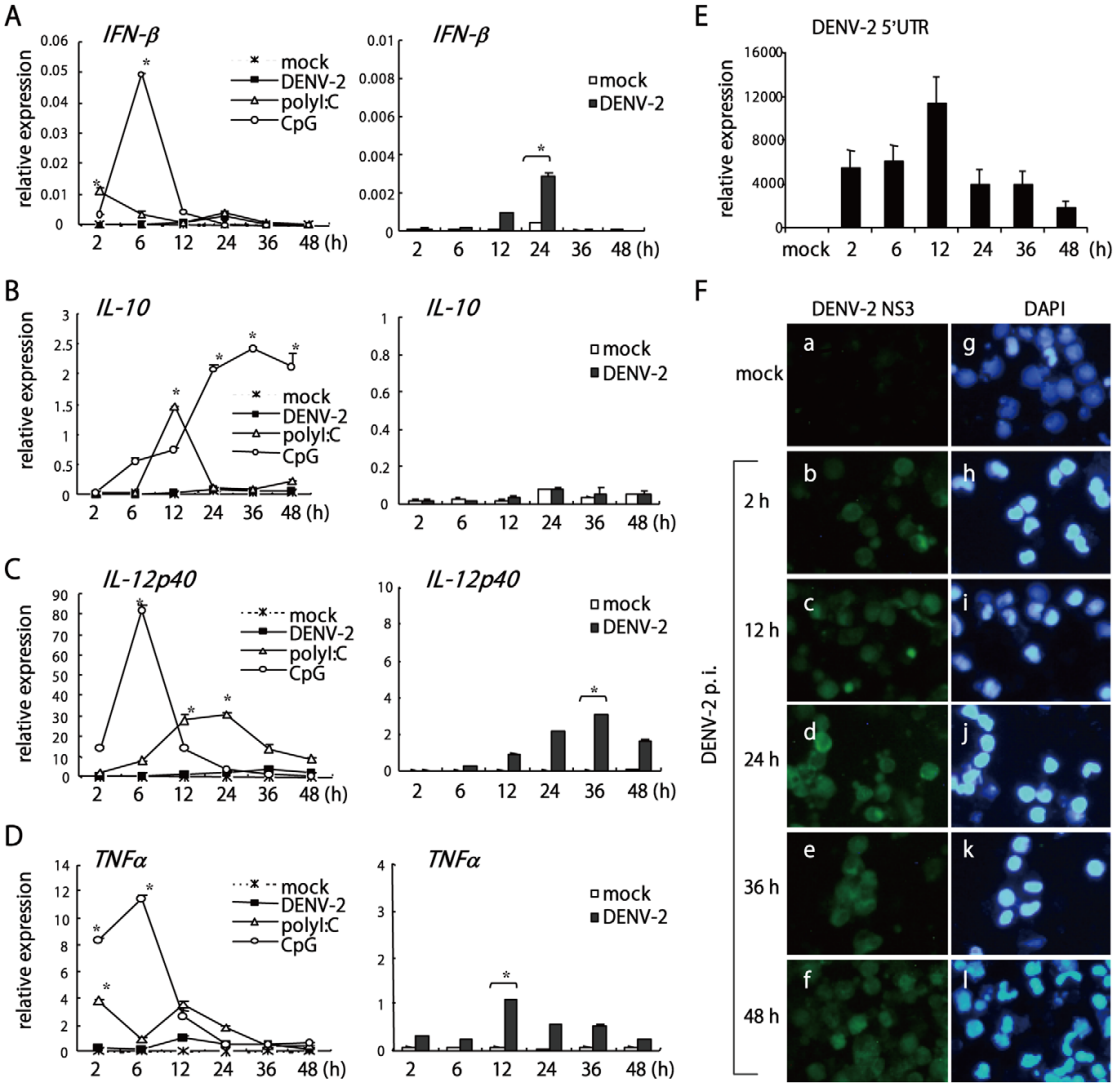
Low levels of IFN-β and cytokine production in DENV-2–infected BMDCs. Bone-marrow–derived dendritic cells (BMDCs) were mock-infected or infected with DENV-2 at a multiplicity of infection (MOI) of 5 or stimulated with the toll-like receptor (TLR) ligands polyI:C (100 µg/ml) or CpG (1 µg/ml), for various times. Quantitative real-time PCR (qPCR) analysis of mRNA levels of IFN-β (**A**), IL-10 (**B**), IL-12p40 (**C**), TNFα (**D**) and DENV-2 viral 5′ UTR RNA (**E**) normalized to the internal control HPRT. Data are mean ±SD of 3 determinations. Data for DENV-2-induced cytokine expression is magnified at the right of each panel. *p≤0.05 compared with mock infection. (**F**) DENV-2-infected BMDCs were immunofluorescently stained with anti-DENV-2 NS3 antibody (green fluorescence, a–f). DAPI staining indicates the location of cell nucleus (blue fluorescence, g–l).

To study the effect of DENV-2 on modulating innate immune response, we determined the levels of type I IFN and cytokines in DENV-2–infected mouse BMDCs by quantitative RT-PCR. The TLR3 ligand polyI:C and the TLR9 ligand CpG greatly stimulated the mRNA expression of IFN-β (124-fold induction at 2 h by polyI:C and 531-fold induction at 6 h by CpG), IL-10 (77-fold at 12 h by polyI:C and 74-fold at 36 h by CpG), IL-12p40 (381-fold at 24 h by polyI:C and 1551-fold at 6 h by CpG), and TNFα (64-fold at 2 h by polyI:C and 180-fold at 6 h by CpG) (Figure 1A–D, left panels). However, levels of IFN-β and these cytokines were much lower in cells with DENV-2 infection (Figure 1, left and right panels): especially, IL-10 was not induced by DENV-2 infection. The kinetic of DENV-2 replication in BMDCs was measured by qPCR with primers specific for DENV-2 5′-UTR (Figure 1E) and by immunofluorescence staining with antibody specific against DENV-2 NS3 (Figure 1F). DENV-2 replication peaked around 12–24 h post infection, in consistence with cytokine induction peaked around 12–36 h post infection. Therefore, infection with DENV-2 inefficiently triggered type I IFN expression and that of other cytokines. Since DENV-2 infection would produce intracellular viral RNA to turn on RLR and TLR signaling cascades for cytokine production, weak induction of DENV-2 for these cytokine genes (Figure 1) implies that DENV-2 may interfere with a common signaling pathway for inducing type I IFN and other inflammatory cytokines.

### DENV-2 infection blocks TLR-triggered IFN-β and IL-10 induction

**Figure 2:**
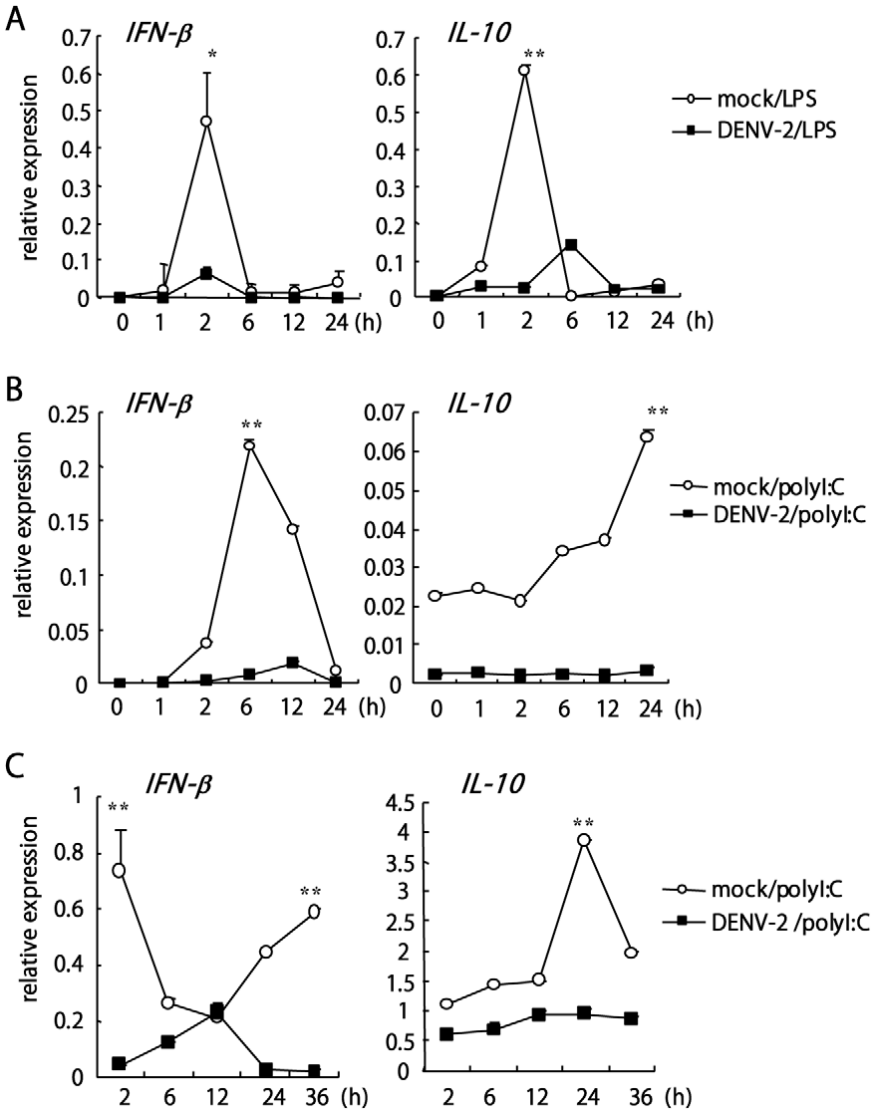
DENV-2 infection blocks TLR-triggered IFN-β and cytokine production. J774A.1 macrophages were mock-infected or infected with DENV-2 (MOI 5) for 24 h before TLR ligand stimulation. qPCR analysis of mRNA expression of IFN-β and IL-10 on cells coincubated with lipopolysaccharide (LPS; 100 ng/ml) (**A**) or polyI:C (100 µg/ml) (**B**) for the indicated times. (**C**) BMDCs were mock-infected or infected with DENV-2 (MOI 5) for 24 h before polyI:C (100 µg/ml) stimulation. qPCR analysis of mRNA expression of IFN-β and IL-10 was analyzed at the indicated times. Normalization was done with the expression level of internal control HPRT. Data are mean±SD of 3 determinations. *, *p*<0.01; **, *p*<0.005.

To determine whether DENV infection could suppress the cytokine induction triggered by TLR signaling, we investigated infection with a murine macrophage cell line J774A.1. Consistent with previous report that J774A.1 is susceptible to DENV infection [27], DENV-2 infection in J774A.1 was shown by immunofluorescence staining with antibody specific against DENV-2 NS3 (Figure S1A), and by qPCR with DENV-2 5′ UTR primers (Figure S1B). J774A.1 cells were mock-infected or infected with DENV-2, then stimulated with LPS or polyI:C. LPS and polyI:C readily promoted the expression of IFN-β (Figure 2A and B; left panels) and IL-10 (Figure 2A and B right panels), but with DENV-2, the expression was diminished. Similar results were noticed in DENV-2-infected BMDCs that polyI:C-activated IFN-β and IL-10 were reduced with DENV-2 infection (Figure 2C). Thus, DENV-2 appears to interfere with IFN-β and cytokine production triggered by TLRs signaling cascade.

### NF-κB activation triggered by TLR ligands is blocked by DENV-2 infection

**Figure 3:**
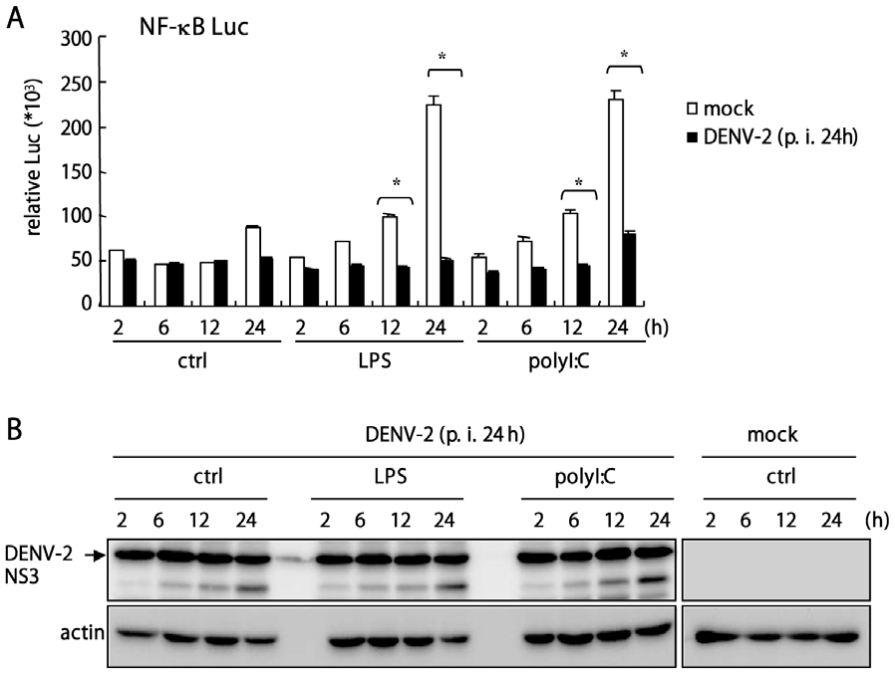
NF-κB activation triggered by LPS and polyI:C is suppressed by DENV-2 infection. (**A**) Dual luciferase assay of reporter activity. A549 cells (1×10^5^) transfected with NF-κB-Luc reporter (0.7 µg) and pRL-TK (0.02 µg), were infected with DENV-2 for 24 h and then were stimulated with LPS or polyI:C (both 1 µg/ml) for various times. Data are mean±SD from 3 determinations. *, *P*≤0.001 (**B**) The cells lysates collected from cells as described in panel A were subjected for immunoblotting assay to detect the expression of DENV-2 NS3 and actin.

**Figure 4:**
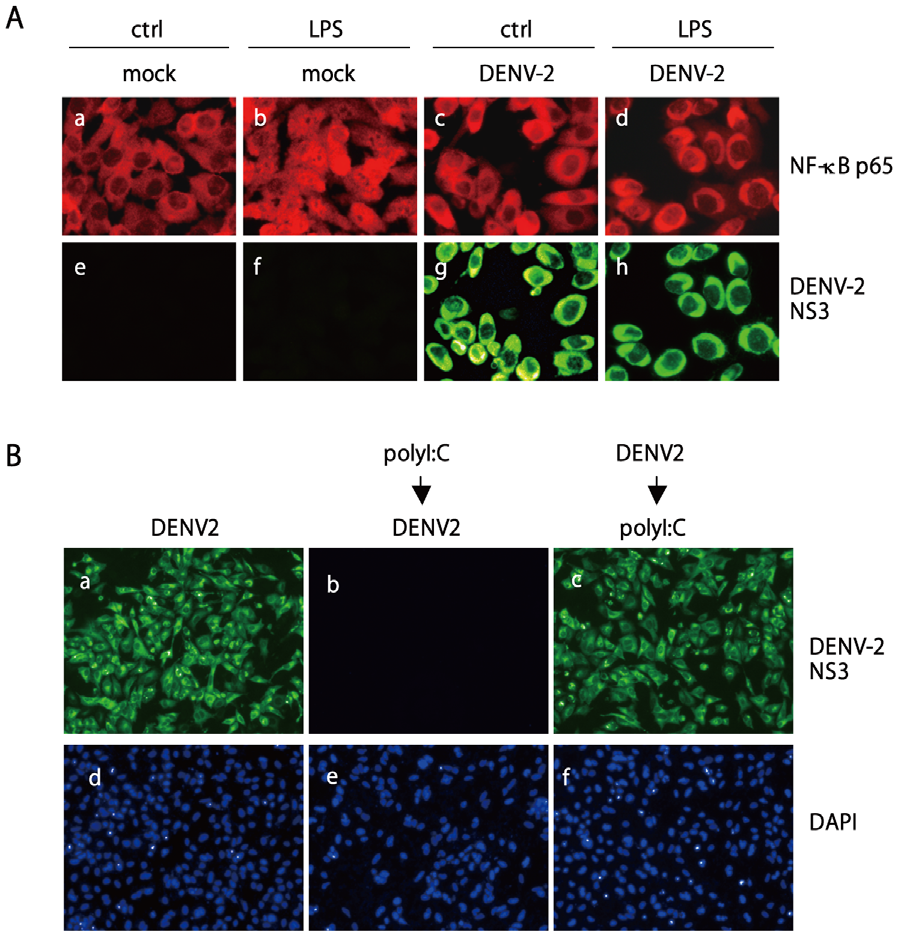
NF-κB p65 nuclear translocation triggered by LPS is blocked by DENV-2 infection. (**A**) Vero cells were infected with DENV-2 for 32 h before stimulation with LPS (1 µg/ml). After 6 h of LPS treatment, the localization of NF-κB p65 was determined by immunostaining with anti-NF-κB p65 antibody (red fluorescence, panels a–d), and DENV-2 infection was determined by anti-DENV-2 NS3 antibody (green fluorescence, panels e–h). Representative cells from the same field are shown for each experimental group. (**B**) A549 cells were infected with DENV-2 for 24 h (panels a and d), or treated with polyI:C (2 µg) by transfection for 24 h prior to DENV-2 infection (panels b and e) or 24 h post DENV-2 infection (panels c and f). DENV-2 infection was monitored by IFA with anti-DENV-2 NS3 antibody (green fluorescence) and the cell nuclei were counter stained by DAPI (blue fluorescence).

NF-κB is an essential molecule in the TLR signaling pathway for inducing type I IFN-β and cytokines [16], [28], [29]. In our infection system, two strains of DENV-2, PL046 and NGC, triggered low degree of NFκB p65 nuclear translocation. Nuclear staining of p65 was noted in 7% and 13% of PL046- and NGC-infected cells, respectively (Figure S2A panels b and c); in contrast, about 80% of the control virus infected cells showed nuclear staining of NF-κB p65 (Figure S2A, panel d). Thus, we checked whether DENV-2 could suppress NF-κB activation triggered by TLRs engagement. With use of a NF-κB-dependent luciferase reporter, NF-κB activation stimulated by the TLR ligands LPS and polyI:C was readily blocked in DENV-2-infected cells at 12 and 24 h post stimulation (Figure 3A). Immunoblotting result of DENV-2 NS3 indicated that the virus was replicating in A549 cells and was not affected by LPS and polyI:C posttreatment (Figure 3B). As well, DENV-2 infection blocked LPS-triggered NF-κB activation in Vero cells as measured by nuclear translocation of NF-κB p65 (Figure 4A); similar results were also observed in J774A.1 macrophages (Figure S2B). Nuclear translocation of NF-kB p65 was slightly increased in A549 cells up to 60 h post DENV-2 infection, but the polyI:C-triggered p65 nuclear translocation was greatly blocked in DENV-2 infected cells (Figure S2C). We also tested the influence of TLR singling in DENV-2 replication, polyI:C pretreatment blocked DENV-2 replication; whereas, the antiviral effect of polyI:C was not seen in cells with established DENV-2 infection (Figure 4B). This data is consistent with previous report [30] and suggest that DENV-2 inhibits TLR signaling to benefit its replication. DENV-2 replication in these two cell lines, A549 and Vero, was confirmed by qPCR with viral 5′ UTR primers and plaque assay for virion production (Figure S3). Thus, DENV-2 downregulates TLR-activated NF-κB, which leads to reduced cytokine expression.

### DENV-2 inhibits ERK1/2 activation

**Figure 5:**
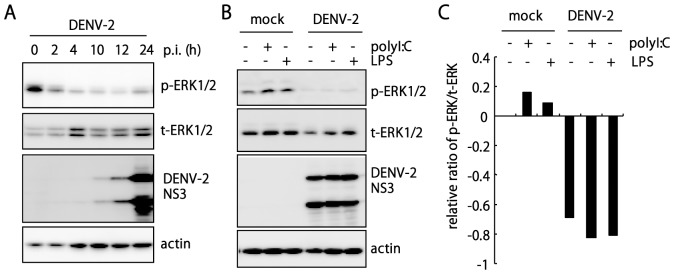
TLR-mediated ERK activation is blocked by DENV-2 infection. (**A**) A549 cells (5×10^5^) were infected with DENV-2 (MOI 5), cell lysates were harvested at the indicated hours post infection (p.i.), and underwent immunoblotting with antibodies (1: 1000 dilution) against phospho-ERK1/2 (p-ERK1/2), total ERK1/2 (t-ERk1/2), DENV-2 NS3, and actin as indicated. (**B**) Immunoblotting analysis of mock- and DENV-2-infected (MOI 5) A549 cells (1×10^6^) stimulated with polyI:C (1 µg/ml) or LPS (1 µg/ml) for 24 h. (**C**) The ratio of phospho-ERK to total ERK in panel B was quantified and calculated with BioSpectrum Image System software (UVP). The mock infection without stimulation is used as the normalization control.

The activity of ERK is associated with the expression of type I IFN and cytokines, particularly IL-10 [31], [32], [33]. We thus further checked whether DENV-2 targets ERK activation by examining the phosphorylated ERK (p-ERK). DENV-2 infection did not activate ERK1/2 phosphorylation and even impaired the basal level of p-ERK1/2 (Figure 5A). Furthermore, polyI:C- and LPS-stimulated phosphorylation of ERK1/2 was decreased with DENV-2 infection (Figure 5B and 5C), so DENV blocks TLR-mediated ERK activation to modulate both arms of the innate immunity response to infection: type I IFN and cytokines.

## Discussion

The pathogenesis mechanism of severe DHF/DSS with DENV infection is not well understood, but evidence suggests that the magnitude of DENV replication and its regulation of innate and adaptive immunity may both contribute [34]. The incidence of DHF/DSS is higher in people with previous exposure to different serotypes of DENV, and antibody-dependent enhancement (ADE) may be a mechanism for DHF/DSS [1]. Enhancing antibodies may increase viral entry and increase the number of infected cells. DENV infection via the ADE route has also been shown to downregulate several genes of the innate immunity system, resulting in suppression of the innate response and increase of DENV replication [35], [36], [37]. In this study, we further demonstrate that DENV per se is a weak cytokine inducer because IL-10, IL-12, and TNFα were induced to a lower extent in DENV-2-infected BMDCs. Furthermore, even in the absence of enhancing antibody, DENV could block NF-κB activation and cytokine induction triggered by TLR signaling. Downregulation of the cytokine system may provide a growth advantage for DENV to propagate in host macrophages and DCs.

IL-10 is a potent immunosuppressor produced by several immune cells [38], and its expression can be triggered by TLR [31], [32]. IL-10 is critical in suppressing excessive inflammation and immunopathologic conditions caused by the host immune system responding to infections [39], [40]. DHF patients showed high serum levels of IL-10, which may be involved in the pathogenesis of severe dengue disease [41], [42]. In a WNV animal infection model, IL-10 downregulated T cell-mediated immunity and had a negative role in antiviral immunity [43]. Thus, our findings that DENV-2 failed to induce IL-10 expression in BMDCs (Figure 1) are unexpected but are consistent with previous reports that IL-10 is not produced from mature human CD1a+ DCs infected with DENV-2 [44], [45] and that microarray data from DENV-1-infected rhesus macaques showed no transcription of IL-10 or other cytokine genes [15]. In addition, we found that DENV-2 could inhibit TLR-triggered IL-10 expression. Therefore, the high IL-10 expression found in patients with dengue-related diseases might not be simply stimulated by the DENV itself. Instead, it may be resulted from uncontrolled DENV replication, which then triggers increased levels of immune activation and increased IL-10 production. DENV infection through the ADE route often induces IL-10 expression, which worsens the host anti-viral system and results in increased DENV production [35], [37], [45]. Thus, DENV infection via ADE or non-ADE routes might have different effects in the immune system.

TLR signaling cascades are mainly controlled by the MyD88-dependent and TRIF-dependent pathways, which both lead to activation of NF-κB and mitogen activated protein kinases (MAPKs) [17], [46]. NF-κB and MAPKs have been suggested to have critical roles in cytokine induction [47], [48]. A recent study showed that constitutive intestinal NF-κB activation does not lead to destructive inflammation unless accompanied by activation of MAPKs such as p38 and ERK [49]. Our results that DENV-2 blocked activation of NF-κB, as well as ERK1/2, support our findings of cytokine production hampered in DENV-2-infected cells. Because both NFκB and ERK1/2 were affected, DENV may suppress an upstream molecular event such as TLR gene expression, as that has been reported for DENV infection through an ADE route [37]. We also detected TLR genes expression in DENV-2 infected J774A.1 macrophages and found that polyI:C-triggered TLR4, TLR5 and TLR13 expression was significantly downregulated by DENV-2 (Figure S4, panels a–c), but not for that of TRL6, TLR7, TLR8 and TLR2 (Figure S4, panels d–g). In contrast, DENV-2 enhanced the gene expression of TLR1 and TLR3 triggered by polyI:C stimulation (Figure S4, panels h–i), suggesting that DENV-2 may modulate certain TLR genes expression in macrophage. Other possibilities, such as whether DENV protease, found to block IFN-β promoter activation [13], may target common molecules involved in type I IFN and cytokine production in TLR signaling, or whether ISG15 that is induced by DENV-2 and functions as an inhibitor of type I IFN production [50] may also contribute to immune evasion, remain to be further studied.

Taiwanese DENV-2 strain PL046 used in this study was isolated from patient with DF, and this virus has been used in the studies of viral pathogenesis and host responses mechanism *in vitro* and *in vivo* [7], [12], [51], [52], [53]. Other groups have reported that DENV-2 strains MON601 (a laboratory strain of NGC) and 16681 downregulate the activation of NF-κB and production of type I IFN and TNFα in human DC, macrophage, and Huh7 cells [14], [54]. A recent report by Chase A. J. et al. [55] also revealed that human DCs infected with several endemic DENV-2 strains failed to polarize the naïve CD4^+^ T cells to effectors, suggesting a defect on T cell priming for DENV-infected DCs [55]. However, one of the strain ARA6894 did not show such kind of inhibition effect, as ARA6894-infected DCs triggered CD4^+^ Th1 polarization with high expression of IFNγ and TNFα. Interestingly, the polyprotein sequences of strain ARA6894 contain nonsynonymous amino acids that are not present in other DENV-2 strains such as PL046, 16681 and NGC [55], suggesting that different impacts on DC's function between these DENV-2 strains might be due to viral genome diversity.

Dengue may be the most important arboviral disease potentially affecting 2 to 3 billion people living in tropical and subtropical areas. DENV mainly infects monocytes, macrophages and DCs that are also the most important innate immune cells. The balance between the protective and pathological immune responses likely contributes to the DENV infection outcomes. Our results demonstrating that DENV can modulate the signaling events triggered by several TLRs are of interest and provide an explanation for how DENV may skew the host immune system.

## Supporting Information

Figure S1 **DENV-2 PL046 replicates in J774A.1 macrophages.** (A) DENV-2-infected J774A.1 cells, MOI of 3 for 24 h, were immunofluorescently stained with anti-DENV-2 NS3 antibody (green fluorescence, panels a and b). DAPI staining indicates the location of cell nucleus (blue fluorescence, panels c and d). (B) J774A.1 (2×10^5^) cells were infected with DENV-2 PL046 (MOI 5) for various times. The levels of DENV-2 viral RNA were measured by qPCR analysis with primers specific for DENV-2 5′-UTR. Data are mean±SD from 3 determinations.
(TIF)

Figure S2 **DENV-2 induces low level of NF-κB activation.** (A) Vero cells were infected with DENV-2 PL046 or NGC, or Japanese encephalitis virus (JEV, strain RP-9) at a MOI of 5 for 24 h. Immunofluorescence analysis showed the subcellular localization of NF-κB p65 (red, panels a–d) and the detection of viral proteins DENV-2 NS3 or JEV NS1 (green, panels e–h). (B) J774A.1 macrophages were infected with DENV-2 PL046 for 48 h before stimulation with LPS (1 µg/ml). After 6 h of LPS treatment, the localization of NF-κB p65 was determined by immunostaining with anti-NF-κB p65 antibody (red fluorescence, panels a–d). DENV-2 infection was determined by anti-DENV-2 NS3 antibody (green fluorescence, panels e–h), and the DAPI presents the nuclear counter stain (blue fluorescence, panels i–l). Representative cells from the same field are shown for each experimental group. (C) A549 cells were infected with DENV-2 PL046 (MOI of 5) for the indicated times. For polyI:C stimulation, DENV-2-infected cells at 36 h p.i. were transfected with polyI:C (2 µg) and incubated for another 24 h, meaning DENV-2 infection for a total of 60 h. For the mock control group, polyI:C stimulation was conducted by polyI:C (2 µg) transfection for 24 h. The immunofluorescence staining of NF-κB P65 was performed as described above, and the DENV-2 infected cells with nuclear p65 were counted. Data are mean±SD from 3 determinations.
(TIF)

Figure S3 **Kinetic analysis of DENV-2 replication in A549 and Vero cells.** A549 and Vero cells were infected with DENV-2 (MOI 5) for various times. (A, C) Total cellular RNA was isolated and detected for the levels of DENV-2 viral RNA by qPCR with primers for DENV-2 5′-UTR. Values represent the average of three assays +/− S.D. (B, D) The culture supernatants were collected for plaque forming assays in BHK-21 cells.
(TIF)

Figure S4 **DENV-2 modulates TLR genes expression.** J774A.1 macrophages were mock-infected or infected with DENV-2 (MOI 5) for 24 h before poly I:C stimulation. TLRs genes expression levels were determined by qPCR analysis on cells with various times of polyI:C (100 µg/ml) stimulation. Normalization was done with the expression level of the internal control HPRT. Values represent the average of three assays +/− S.D. * *p*<0.01. The qPCR primers for TLRs are listed in Table S1.
(TIF)

Table S1 **qPCR primer sequences for mouse TLRs genes.**
(DOC)
