# Supplementary figures and images for: Dengue Virus Serotype 2 Blocks Extracellular Signal-Regulated Kinase and Nuclear Factor-κB Activation to Downregulate Cytokine Production
Source: PLoS One. 2012 Aug 22;7(8):e41635. doi: 10.1371/journal.pone.0041635 (PMC3425550; doi:10.1371/journal.pone.0041635)

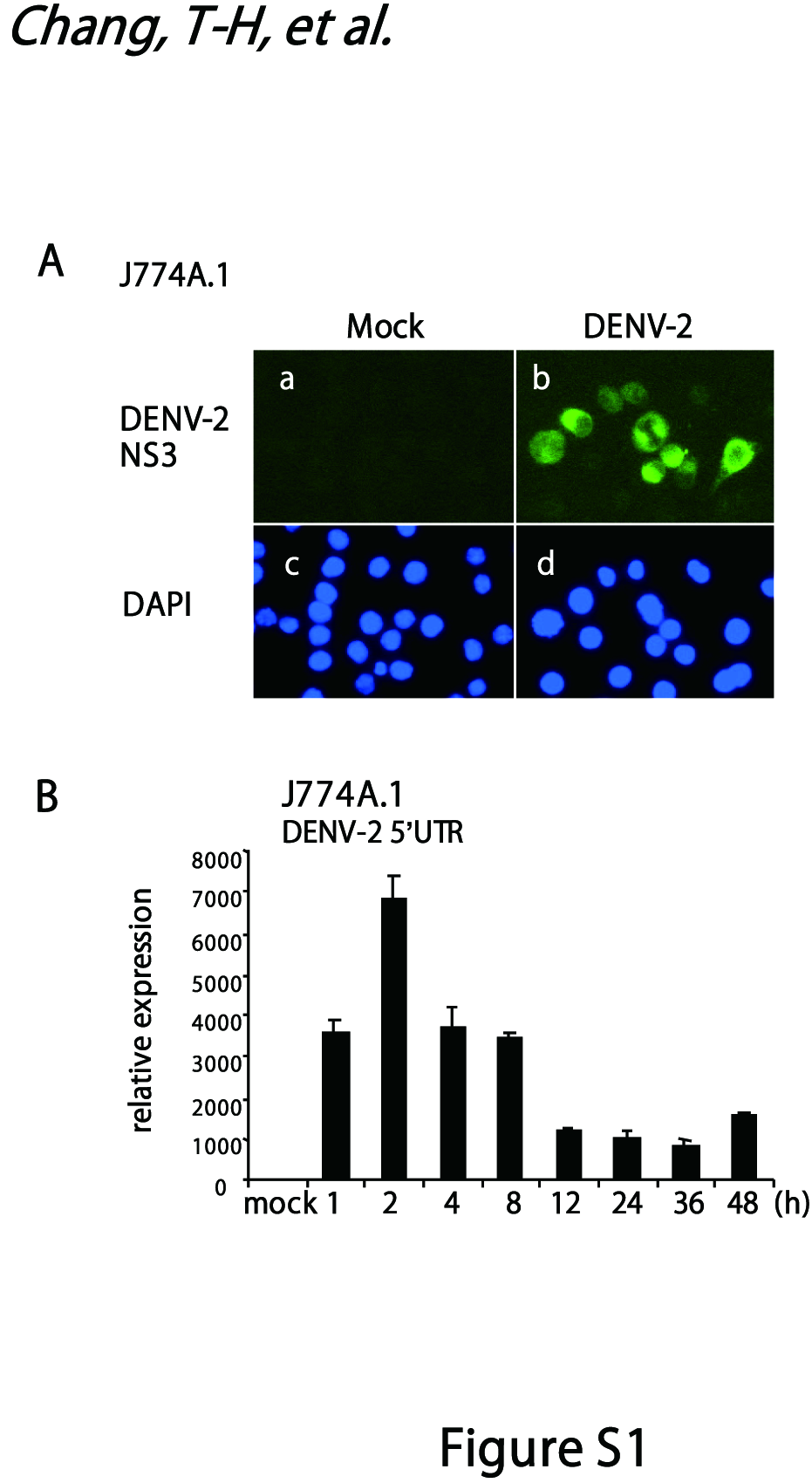

Supplement: Figure S1 — DENV-2 PL046 replicates in J774A.1 macrophages. (A) DENV-2-infected J774A.1 cells, MOI of 3 for 24 h, were immunofluorescently stained with anti-DENV-2 NS3 antibody (green fluorescence, panels a and b). DAPI staining indicates the location of cell nucleus (blue fluorescence, panels c and d). (B) J774A.1 (2×10^5^) cells were infected with DENV-2 PL046 (MOI 5) for various times. The levels of DENV-2 viral RNA were measured by qPCR analysis with primers specific for DENV-2 5′-UTR. Data are mean±SD from 3 determinations. (TIF) [file pone.0041635.s001.tif]

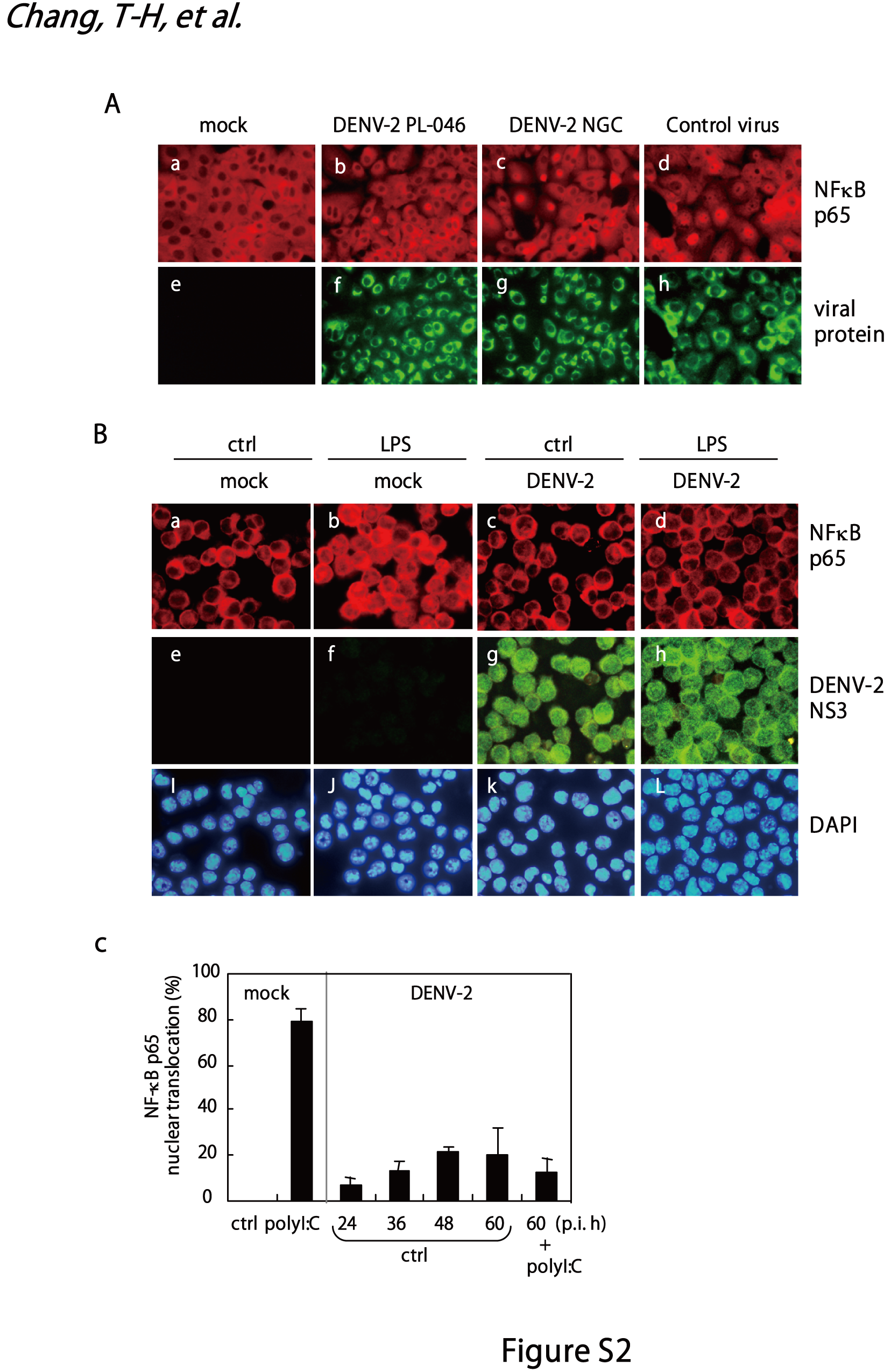

Supplement: Figure S2 — DENV-2 induces low level of NF-κB activation. (A) Vero cells were infected with DENV-2 PL046 or NGC, or Japanese encephalitis virus (JEV, strain RP-9) at a MOI of 5 for 24 h. Immunofluorescence analysis showed the subcellular localization of NF-κB p65 (red, panels a–d) and the detection of viral proteins DENV-2 NS3 or JEV NS1 (green, panels e–h). (B) J774A.1 macrophages were infected with DENV-2 PL046 for 48 h before stimulation with LPS (1 µg/ml). After 6 h of LPS treatment, the localization of NF-κB p65 was determined by immunostaining with anti-NF-κB p65 antibody (red fluorescence, panels a–d). DENV-2 infection was determined by anti-DENV-2 NS3 antibody (green fluorescence, panels e–h), and the DAPI presents the nuclear counter stain (blue fluorescence, panels i–l). Representative cells from the same field are shown for each experimental group. (C) A549 cells were infected with DENV-2 PL046 (MOI of 5) for the indicated times. For polyI:C stimulation, DENV-2-infected cells at 36 h p.i. were transfected with polyI:C (2 µg) and incubated for another 24 h, meaning DENV-2 infection for a total of 60 h. For the mock control group, polyI:C stimulation was conducted by polyI:C (2 µg) transfection for 24 h. The immunofluorescence staining of NF-κB P65 was performed as described above, and the DENV-2 infected cells with nuclear p65 were counted. Data are mean±SD from 3 determinations. (TIF) [file pone.0041635.s002.tif]

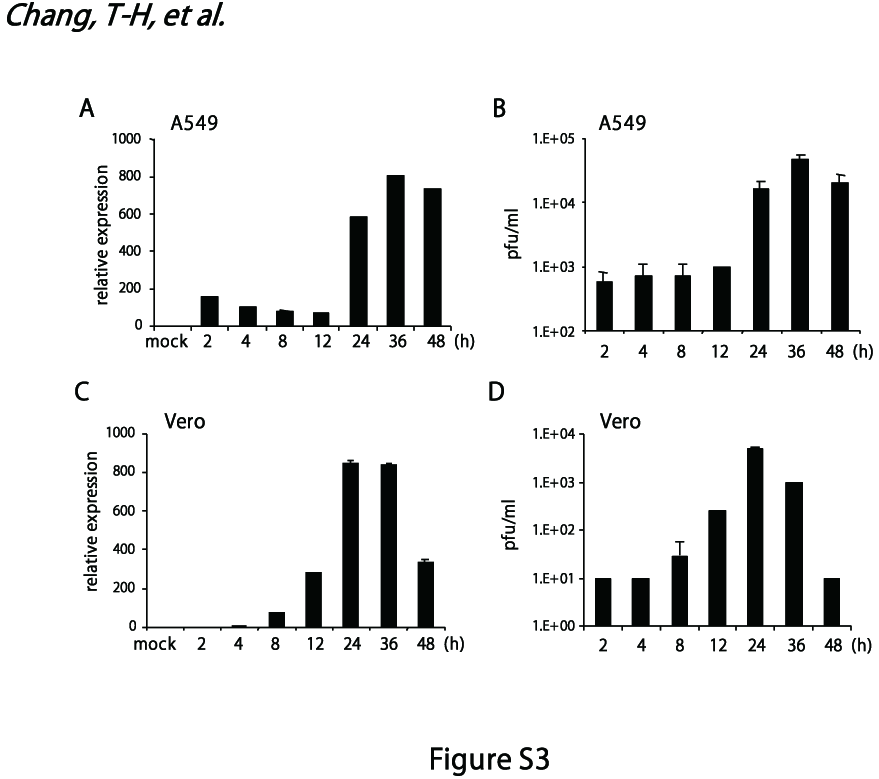

Supplement: Figure S3 — Kinetic analysis of DENV-2 replication in A549 and Vero cells. A549 and Vero cells were infected with DENV-2 (MOI 5) for various times. (A, C) Total cellular RNA was isolated and detected for the levels of DENV-2 viral RNA by qPCR with primers for DENV-2 5′-UTR. Values represent the average of three assays +/− S.D. (B, D) The culture supernatants were collected for plaque forming assays in BHK-21 cells. (TIF) [file pone.0041635.s003.tif]

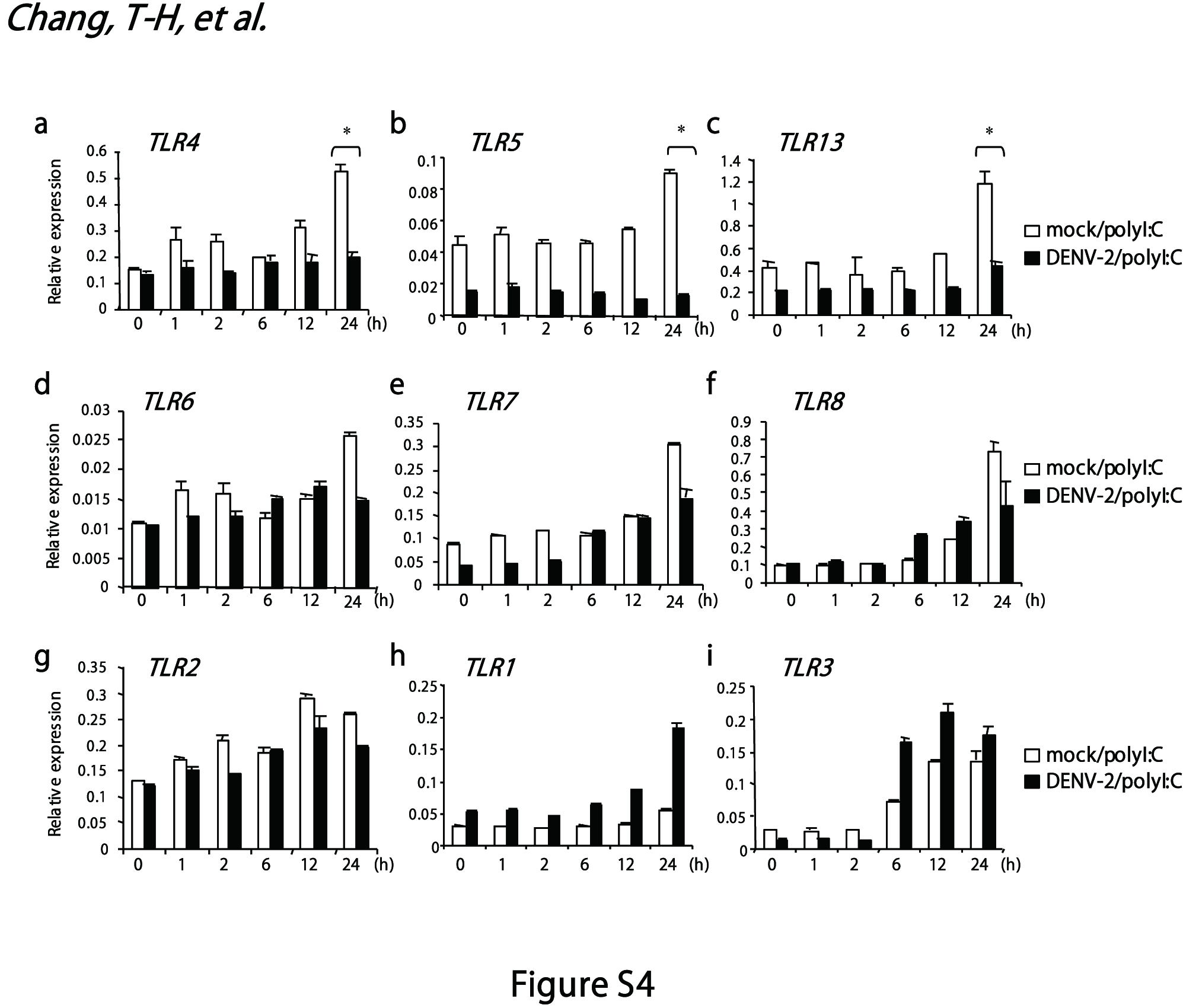

Supplement: Figure S4 — DENV-2 modulates TLR genes expression. J774A.1 macrophages were mock-infected or infected with DENV-2 (MOI 5) for 24 h before poly I:C stimulation. TLRs genes expression levels were determined by qPCR analysis on cells with various times of polyI:C (100 µg/ml) stimulation. Normalization was done with the expression level of the internal control HPRT. Values represent the average of three assays +/− S.D. * p<0.01. The qPCR primers for TLRs are listed in Table S1. (TIF) [file pone.0041635.s004.tif]
