# Supplementary material for: Dengue Virus Serotype 2 Blocks Extracellular Signal-Regulated Kinase and Nuclear Factor-κB Activation to Downregulate Cytokine Production
Source: PLoS One. 2012 Aug 22;7(8):e41635. doi: 10.1371/journal.pone.0041635 (PMC3425550; doi:10.1371/journal.pone.0041635)
Supplement: Table S1 — qPCR primer sequences for mouse TLRs genes. (DOC) [file pone.0041635.s005.doc]

| **Gene** | Accession number | **Primer sequences** |
| --- | --- | --- |
| Tlr1 | NM_030682 | F’-GGCTTTGCAGGAACTCAATGTAG  R’-CCCCGCACCCAGGAA |
| Tlr2 | NM_011905 | F’-CACCACTGCCCGTAGATGAA  R’-GCCTCGGAATGCCAGCTT |
| Tlr3 | NM_126166 | F’-CCCAATGGAAGAACAAGACCAA  R’-AAGGACGCCTGCTTCAAAGTC |
| Tlr4 | NM_021297 | F’-GCAGCAGGTGGAATTGTATCG  R’-TGTGCCTCCCCAGAGGATT |
| Tlr5 | NM_016928 | F’-TCACCTGCCATTGATGTCCTT  R’-ACGCAATAGGATGGAGGGAAA |
| Tlr6 | NM_011604 | F’-GACCTGCCACCAAGAACAAAA  R’-GGCATCCGAAGCTCAGATATAGA |
| Tlr7 | NM_133211 | F’-GATGTTATAATGTCCCATATCCGTGTAC  R’-AAGCATTGTCATGGATCTGTAAGG |
| Tlr8 | NM_133212 | F’- TCTATTCTGGAGCTACATGGGAACTA  R’-TCATCTAGCCAGCTTCGAAAATC |
| Tlr13 | NM_205820 | F’-ACAAAGACACGGGATTCAGGTT  R’-TCCTGCAAACTACCCAATCCTT |

Accession number of measured genes: *Ifnb* NM_010510, *Il10* NM_010548, *Tnfa* NM_013693, *Il12p40* NM_009352.2, *Hprt* NM_013556, *DENV-2 PL046* AJ968413.
